# Photocatalytic Microporous Membrane against the Increasing Problem of Water Emerging Pollutants

**DOI:** 10.3390/ma12101649

**Published:** 2019-05-21

**Authors:** Pedro M. Martins, Joana M. Ribeiro, Sara Teixeira, Dmitri. Y. Petrovykh, Gianaurelio Cuniberti, Luciana Pereira, Senentxu Lanceros-Méndez

**Affiliations:** 1 Centre of Physics, University of Minho, 4710-057 Braga, Portugal; joanaribeiro93@hotmail.com; 2 Department of Biological Engineering, University of Minho, 4710-057 Braga, Portugal; lucianapereira@deb.uminho.pt; 3 Institute of Science and Innovation for Bio-Sustainability (IB-S), University of Minho, 4710-057 Braga, Portugal; 4 Institute for Materials Science and Max Bergmann Center of Biomaterials, TU Dresden, 01062 Dresden, Germany; sara.teixeira@nano.tu-dresden.de (S.T.); g.cuniberti@tu-dresden.de (G.C.); 5 International Iberian Nanotechnology Laboratory, Avenida Mestre José Veiga, 4715-330 Braga, Portugal; dmitri.petrovykh@inl.int; 6 Dresden Center for Computational Materials Science, TU Dresden, 01062 Dresden, Germany; 7 Center for Advancing Electronics Dresden, TU Dresden, 01062 Dresden, Germany; 8 Basque Center for Materials, Applications, and Nanostructures, UPV/EHU Science Park, 48940 Leioa, Spain; 9 IKERBASQUE, Basque Foundation for Science, 48013 Bilbao, Spain

**Keywords:** immobilization, pharmaceuticals, photocatalysis, PVDF-TrFE, titanium dioxide

## Abstract

Emerging pollutants are an essential class of recalcitrant contaminants that are not eliminated from water after conventional treatment. Here, a photocatalytic microporous membrane based on polyvinylidene difluoride-co-trifluoroethylene (PVDF−TrFE) with immobilised TiO_2_ nanoparticles, prepared by solvent casting, was tested against representative emerging pollutants. The structure and composition of these polymeric membranes were characterized by scanning electron microscopy, energy dispersive X-ray spectroscopy, Fourier-transform infrared spectroscopy, porosimetry, and contact angle goniometry. The nanocomposites exhibited a porous structure with a uniform distribution of TiO_2_ nanoparticles. The addition of TiO_2_ did not change the structure of the polymeric matrix; however, it increased the wettability of the nanocomposite. The nanocomposites degraded 99% of methylene blue (MB), 95% of ciprofloxacin (CIP), and 48% of ibuprofen (IBP). The microporous nanocomposite exhibited no photocatalytic efficiency loss after four use cycles, corresponding to 20 h of UV irradiation. The reusability of this system confirms the promising nature of polymer nanocomposites as the basis for cost-effective and scalable treatments of emerging pollutants.

## 1. Introduction

Emerging pollutants are increasingly recognized as critical environmental contaminants [1] that usually appear at low concentrations (ng L^−1^ to μg L^−1^) and have been detected in both wastewater and drinking water [2,3]. Emerging pollutants include pharmaceuticals, personal care products, endocrine-disrupting compounds, and pesticides [4,5]. While the environmental effects of these compounds are as yet unclear, they are potentially harmful to humans and other organisms [6,7]. 

Pharmaceuticals are a particularly critical class of anthropogenic emerging pollutants present in water [4,8]. These pollutants, including anticonvulsants, antidepressants, and beta-blockers [9], are transported via sewer networks to wastewater treatment plants [10,11,12], where such compounds are not treated effectively [9,13,14,15] and ultimately discharged, contaminating the surface, ground, and drinking water [10,16]. Accordingly, an increasing interest in advanced oxidation processes (AOPs) [17,18] is motivated by their potential to enhance mitigation of water pollution [19].

The AOP process requires a photocatalyst that, under illumination, produces reactive hydroxyl radicals [20], which can degrade organic contaminants [21,22] via oxidation to carbon dioxide, water, and inorganic compounds [23,24,25,26]. Titanium dioxide is well established as an active and reusable photocatalyst for applications in environmental remediation [17,26]. The practical advantages of the inherently simple recovery and re-utilisation of the photocatalyst are essential considerations behind the use of supported nanoparticles [11,27], with immobilisation methods optimised to increase their surface density [28,29] and to limit the loss of the overall photocatalytic efficiency [11,17,21].

Among various options for support materials—which include glass [30], stainless steel [31,32], perlite [30], and optical fibres [33,34]—polymers [28,35,36,37,38,39] are widely used because they are inert, inexpensive, mechanically stable, and durable [40,41,42,43]. In particular, polyvinylidene difluoride-co-trifluoroethylene (PVDF−TrFE) exhibits excellent chemical, mechanical, thermal, and UV resistance, attributed to the stable C–F bonds of the polymer chain [28,44]. Regarding morphology and microstructure, PVDF−TrFE can be produced, having controlled porosity [28,29,44], as films [45], fibers [37,46,47,48], or membranes [28,29,49,50].

Nanocomposites of TiO_2_ nanoparticles supported on PVDF-TrFE (TiO_2_/PVDF-TrFE) have previously demonstrated photocatalytic activity in the degradation of methylene blue (MB) [28]. In this work, the preparation of TiO_2_/PVDF-TrFE nanocomposites has been optimised to lower the photocatalyst content and to improve the characteristics of the nanocomposite at the microstructure level and to prove and understand the reusability nature of these materials. The efficiency and reusability of the optimised TiO_2_/PVDF-TrFE microporous nanocomposite were then tested in the degradation of representative emerging pollutants [1,7,15]: An antibiotic, ciprofloxacin (CIP) [10,15,51,52,53], and an anti-inflammatory, ibuprofen (IBP).

## 2. Materials and Methods

### 2.1. Materials and Reagents

PVDF–TrFE (70:30) was obtained from Solvay (Brussels, Belgium). P25^®^-TiO_2_ nanoparticles was kindly supplied by EVONIK (Essen, Germany). MB and *N*,*N*-dimethylformamide (DMF) 99% were supplied by Merck. CIP 98% and IBP 98% were obtained from Sigma-Aldrich (Darmstadt, Germany). NaOH 97% was obtained from VWR (Radnor, PA, USA). Milli-Q ultrapure water was used in all experiments.

### 2.2. Nanocomposites Production

Nanocomposites of wt. 8% in TiO_2_/PVDF-TrFE matrix were produced by solvent casting. For this purpose, 0.087 g of P25 TiO_2_ nanoparticles and 9 mL of DMF were placed in an ultrasonication bath for 3 h to achieve a good nanoparticles dispersion. Later, 1 g of PVDF-TrFE was added to the solution and stirred for 2 h, until complete dissolution had been achieved. The solution was then poured into a glass Petri dish for solvent evaporation, approximately 4–5 days at room temperature.

### 2.3. Nanocomposites Characterisation

The morphology of the microporous nanocomposites, before and after four photocatalytic uses, was assessed by scanning electron microscopy (SEM). The samples were coated for 30 s with a thin gold layer and analysed with a Quanta 650 SEM (Thermo Fisher, Hillsboro, OR, USA). Pore size measurements were performed for each sample, measuring 50 pore diameters using Image J software (1.50i, National Institutes of Health, Bethesda, MD, USA). Energy dispersive X-ray spectroscopy (EDX) was performed with an INCA 350 spectrometer (Oxford Instruments NanoAnalysis & Asylum Research, High Wycombe, UK), also before and after four photocatalytic uses, but without application of the gold coating. 

A pycnometer was used to assess the porosity of nanocomposites. A 25 mL pycnometer was filled with ethanol until the limit and weighed. A nanocomposite sample of a known mass was then inserted in the pycnometer, which was again filled with ethanol until the saturation limit was reached. The nanocomposite sample was then removed with tweezers, and the pycnometer with the remaining ethanol was weighed. This procedure was repeated three times for each sample; the measured values are presented in this work as the average and the standard deviation. The porosity ϕ (%) can be calculated using Equation (1):

(1) $$\phi =\frac{m_{2}-m_{3}-m_{s}}{m_{1}-m_{3}}$$

where *m*_s_ is the mass of the sample, *m*_1_ is the mass of the pycnometer, *m*_2_ is the mass of the pycnometer filled with ethanol and the sample, and *m*_3_ is the mass of the pycnometer with the ethanol remaining after withdrawing the sample [29,50].

The wettability of nanocomposites was characterised by contact-angle goniometry, using a Data Physics SCA20 microscope (DataPhysics Instruments GmbH, Filderstat, Germany). Three droplets of 3 µL of distilled water were deposited on different sites of the films using a microsyringe, at a drop rate of 5 µL s^−1^ and temperature of ca. 20 °C. The water contact angle was measured immediately after the drop deposition and after 10 min. The half-angle algorithm was applied by the software to calculate the contact angle values as the mean of the right and left angles measured for each drop. The same measurements were repeated after the samples had been exposed to UV light for 30 min. 

Fourier-transform infrared spectroscopy (FTIR) in the attenuated total reflectance (ATR) mode was used to assess the changes in the chemistry of nanocomposites qualitatively. FTIR-ATR was performed on the nanocomposites before and after four cycles of photocatalytic degradation of MB. The spectra were obtained using an FTIR Alpha (Bruker Corporation, Billerica, MA, USA) instrument over a range of 650–4000 cm^−1^ using 64 scans with a resolution of 4 cm^−1^.

### 2.4. Photocatalytic Degradation

The 2.5 × 10 cm^2^ nanocomposite films were fixed by double-sided tape to the inner side of a beaker that was filled with 50 mL of aqueous solutions of 2 mg L^−1^ of MB, 5 mg L^−1^ of CIP, or 15 mg L^−1^ of IBP. These concentrations were chosen to start each measurement at approximately the same absorbance value of ca. 0.6, thus ensuring a similar dynamic range for all the measurements. To reach the adsorption–desorption equilibrium, the solutions (MB, CIP, and IBP) and nanocomposite films were kept in the dark (wrapped in aluminium foil) under stirring for 30 min.

In each photocatalytic degradation cycle, the beakers containing the solutions and microporous nanocomposite were illuminated from 15 cm by a device that produces UV radiation with a peak wavelength at 365 nm (6 Philips 8W mercury fluorescent lamps, UMEX). A UV34 Lux Meter (PCE) was used to monitor and maintain the UV intensity within a range of 1.8–1.9 mW cm^−2^. The photocatalytic degradation was carried out in quadruplicate under the same experimental conditions. Before reusing after each cycle, the nanocomposite films remained fixed onto the inner side of the beakers and were cleaned with Millipore water under magnetic stirring for 5 min, followed by air drying at room temperature.

The photodegradation rate was determined by monitoring the intensity variation of the main absorption peak of each compound (MB (λ_max_ = 665 nm), CIP (λ_max_ = 280 nm), and IBP (λ_max_ = 262 nm) in the witness samples collected at discrete time intervals throughout each degradation cycle. The absorbance spectra in the range of 200–800 nm were acquired using a CARY-100 UV-vis spectrophotometer (Varian). The quantification of photocatalytic degradation is based on the Langmuir–Hinshelwood model (Equation (2)):

(2) $$\ln\left(\frac{C}{C_{0}}\right)=-kt$$

where *C* and *C*_0_ represent, respectively, the concentration of the compound at time *t* and *t* = 0 (initial) obtained from the maximum value of optical absorbance, and *k* is the pseudo-first-order reaction rate constant (min^−1^) [50,54].

## 3. Results and Discussion

### 3.1. Nanocomposite Characterisation

**Figure 1:**
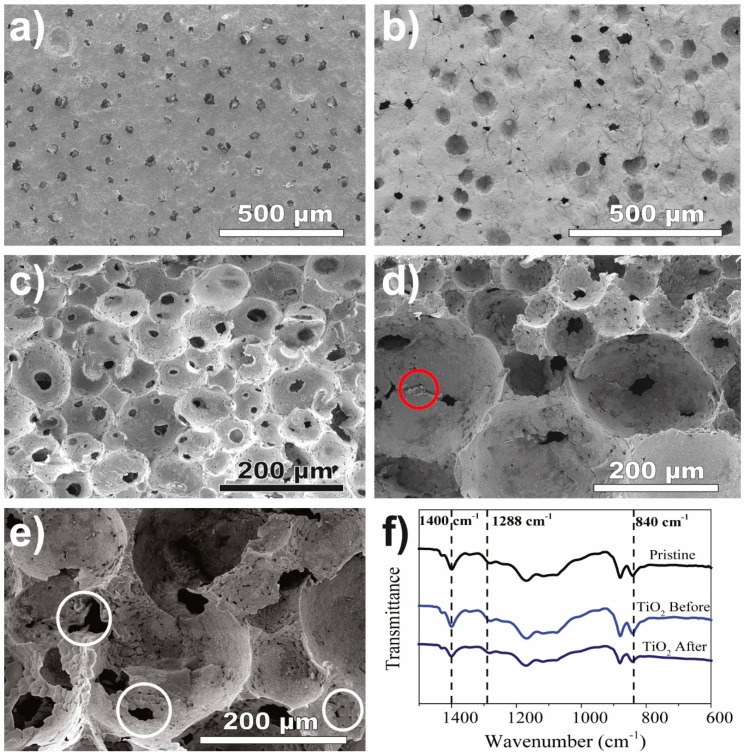
SEM surface of TiO_2_/PVDF-TrFE before (**a**) and after four MB degradation assays (**b**). Pristine (**c**) and TiO_2_/PVDF-TrFE before use (**d**) and TiO_2_/PVDF-TrFE after four MB degradations (**e**). The red circles indicate the particles within the pores and white circles indicate small pores inside the pores (pores interconnectivity); (**f**) FTIR-ATR spectra before and after four MB degradations using the nanocomposites. PVDF-TrFE, polyvinylidene difluoride-co-trifluoroethylene; methylene blue, MB; ATR, attenuated total reflectance.

The surface SEM images of TiO_2_/PVDF-TrFE before use (Figure 1a) reveal the presence of pores and small pores inside the pores, allowing pore interconnectivity and the percolation of polluted solutions through the microporous structure. It was found that even after four MB degradation assays (Figure 1b), the structure and porosity remained unchanged. 

The microporous structure of the pristine PVDF-TrFE (Figure 1c) and the TiO_2_/PVDF-TrFE nanocomposite sample before and after four photocatalytic cycles (Figure 1d,e, respectively) is revealed in the cross-section SEM images. All the samples exhibit spherical pores and high pore interconnectivity, characterised by the presence of smaller pores inside the larger pores (white circles in Figure 1e). The resilient attachment of the TiO_2_ nanoparticles to the polymeric matrix is confirmed by observing the particles both before and after photodegradation measurements (red circles in Figure 1d,e). It is evident, and by comparison with Figure 1d, the TiO_2_ nanoparticles after use are less aggregated than before use. Although SEM inspection shows the presence of pores and TiO_2_ nanoparticles, both for surface and cross-section images, the nanocomposite cross-section reveals a more prominent amount of particles and pores. 

The incorporation of TiO_2_ nanoparticles into the PVDF-TrFE matrix does not significantly change the porosity of the material, which was estimated using a pycnometer to be 81 ± 6% and 74 ± 3% for the pristine PVDF-TrFE and TiO_2_/PVDF-TrFE nanocomposite samples, respectively. The microstructure of the pores, however, does undergo some changes: the pore size distribution broadens from a 10–130 µm range in the pristine PVDF-TrFE to a 25–325 µm range in the TiO_2_/PVDF-TrFE nanocomposite and then narrows to a 5–65 µm range after four photocatalytic cycles. The average pore sizes for all three samples in Figure 1 are not significantly different because of the widths of the respective distributions; however, the trend of the average value is expected, following the increase and decrease of the overall range. The measured values of porosity and pore sizes are in good agreement with the literature [28,29,50] and lie within ranges that are considered beneficial for photocatalytic applications. The high interconnectivity of the pores observed in SEM images is also expected to enhance the penetration of light to the photocatalysts and the mass transfer of pollutants and reactants throughout the nanocomposite simultaneously [17,30,51].

The FTIR-ATR spectra in Figure 1d confirm that the PVDF-TrFE matrix crystallises in the β-phase, with or without nanoparticles present, and that the β-phase is maintained in the microporous nanocomposites after four photocatalytic cycles. The characteristic bands of the PVDF-TrFE polymer in the β-phase are present in all three analysed samples and appear at 840, 1279–1290, and 1400 cm^−1^. No bands attributed to the nonpolar α-phase (766, 795, 855, and 976 cm^−1^) or the γ-phase (776, 812, 833, and 1234 cm^−1^) were observed. The chemical structure of the nanocomposites is not qualitatively affected after four photocatalytic cycles under UV illumination, which is in agreement with previous reports that PVDF copolymers are stable under UV radiation [28,29,50] and thus, are suitable as supports for photocatalysts.

**Figure 2:**
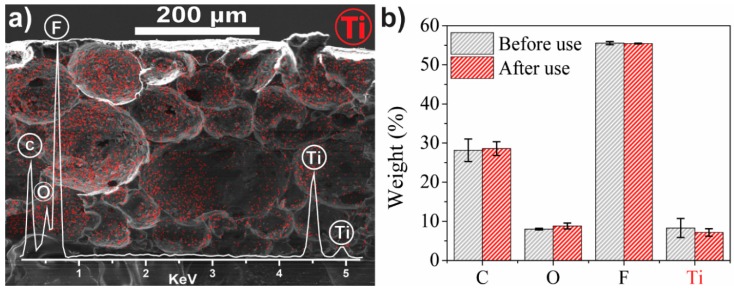
(**a**) SEM-EDX mapping image of the presence and distribution of Ti (red) in the PVDF-TrFE matrix and inset of the EDX spectrum with the identification of the detected elements. (**b**) Elemental percentages of Ti, O, F, and C before and after use.

The retention of TiO_2_ nanoparticles in TiO_2_/PVDF-TrFE nanocomposites after four cycles of photodegradation of MB is confirmed by the stable elemental composition measured by EDX (Figure 2): carbon, oxygen, fluoride and titanium, which were identified both before and after four photocatalytic cycles of MB degradation; the presence of C and F can be attributed to the PVDF-TrFE matrix and of Ti and O—to TiO_2_ nanoparticles. The measured F/C and O/Ti ratios deviate from those expected based on the nominal stoichiometry (2 vs. 1–1.5 and 1 vs. 2, respectively), which is not unexpected for samples with a highly heterogeneous microstructure (Figure 2a). Accordingly, the absolute values of the measured elemental composition cannot be considered to be reliable; however, given the similarity of the overall microstructure of TiO_2_/PVDF-TrFE nanocomposites before and after photodegradation cycles (Figure 1b,c), relative elemental fractions can be compared quantitatively. There are no statistically significant differences in the elemental fractions before and after the photodegradation cycles (Figure 2b), indicating that there is no significant loss of the TiO_2_ nanoparticles from the nanocomposite film. Furthermore, EDX mapping confirms the excellent dispersion of TiO_2_ nanoparticles throughout the porous microstructure of the nanocomposite film (red dots in Figure 2a). 

The weight % of each element before and after use is presented in Figure 2b: carbon (28.2 ± 2.9 wt%) and fluorine (55.5 ± 0.4 wt%) indicate the presence of PVDF-TrFE while titanium (8.3 ± 2.4 wt%) and oxygen (8.0 ± 0.2 wt%) correspond to the nanoparticles. The retention of TiO_2_ nanoparticles after four use cycles is indicated by the lack of significant differences in the Ti and O concentrations, respectively, 7.2 ± 1.0 and 8.8 ± 1.2 wt%, after four use cycles. The stable composition supports the conclusions from the SEM images regarding the efficiency of the attachment between TiO_2_ nanoparticles and the polymer matrix. The presence of TiO_2_ nanoparticles and their excellent dispersion throughout the porous microstructure, after four use cycles, is indicated by the Ti signatures in EDX mapping (red dots in Figure 2a). The presence of TiO_2_ nanoparticles inside the pores supports the remarkable reusability displayed in the photocatalytic assays, confirming the reduced loss of nanoparticles through leaching. 

**Figure 3:**
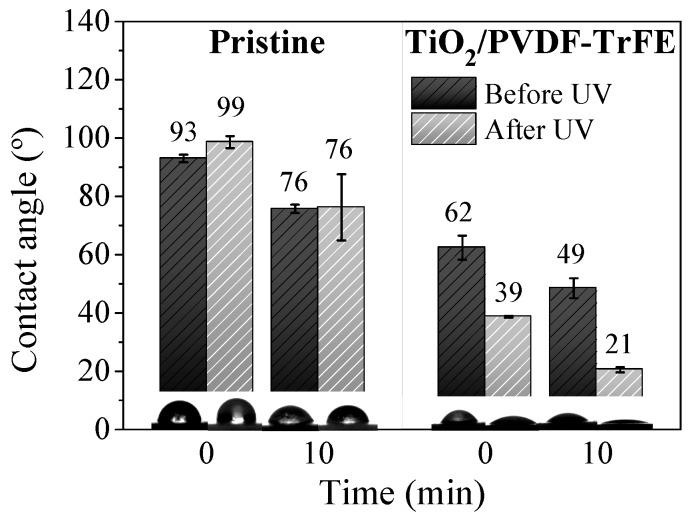
Average water contact angles of the pristine and TiO_2_/PVDF-TrFE membranes, before and after exposure to UV radiation for 30 min.

The pristine membranes exhibited water contact angles of ≈ 93° and 76° after 0 and 10 min of the deposition of the drops, respectively (Figure 3). After exposure to UV radiation, the contact angles did not change significantly for both measured times, which is consistent with the well-established UV resistance of fluorinated polymers [54,55]. The sample did not become more hydrophilic, indicating that the fluorinated functional groups did not break down under UV radiation [56,57]. In contrast to the hydrophobic pristine polymer, the nanocomposites exhibited a significantly more hydrophilic behavior both before (water contact angles of 62° and 49° for 0 and 10 min of droplet deposition, respectively) and after the UV exposure (water contact angles of 39° and 21° for 0 and 10 min of droplet deposition, respectively). The super-hydrophilic properties of TiO_2_ after UV exposure are responsible for this reduction in the contact angles [58]. The enhanced wettability of the TiO_2_/PVDF-TrFE nanocomposites is essential for improving the interaction between the pollutants and the TiO_2_ nanoparticles, favouring the adsorption process that is critical for efficient photocatalysis. Both the hydrophilicity (Figure 3) and highly porous structure (Figure 1) of the nanocomposites should help to mitigate the mass transfer limitations associated with the immobilisation of photocatalyst nanoparticles.

### 3.2. Photocatalytic Degradation

**Figure 4:**
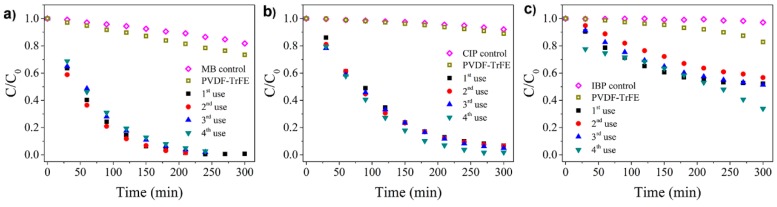
Decrease of the content of (**a**) MB, (**b**) CIP, and (**c**) IBP during four cycles of photocatalytic treatment by 8% TiO_2_/PVDF-TrFE sample under UV irradiation. CIP, ciprofloxacin; IBP, ibuprofen.

The photocatalytic performance of the TiO_2_/PVDF-TrFE nanocomposite was tested in the degradation of MB, CIP, and IBP, under UV irradiation. The photocatalytic degradation curve for four use cycles was measured for each of the three pollutants (Figure 4), monitoring the respective absorbance peaks. As controls, the photocatalytic activity was also tested for the pristine PVDF-TrFE membranes and solutions of each pollutant in the absence of any membrane. For both types of controls, only minimal (when any) photodegradation was observed, confirming that the UV exposure in the absence of the photocatalyst does not induce the degradation process. The adsorption of the three pollutants to the TiO_2_/PVDF-TrFE nanocomposite in the dark (without UV radiation) for 300 min is negligible, as shown in the Supplementary Information (Figure S1). 

The reference MB solution was completely degraded in the presence of the photocatalytic nanocomposite (Figure 4a), becoming colourless after ca. 240 min of irradiation. A higher weight fraction (15 vs. 8% wt.) of TiO_2_ had been tested previously [28], resulting in a lower photocatalytic activity for this reference compound. 

The increased activity of the TiO_2_/PVDF-TrFE nanocomposite demonstrated in Figure 4a can be attributed to the larger pore size (initial average size of 80 ± 68 µm vs. 21 ± 8 µm in) [28], which is expected to increase water percolation and thus promote the contact between the pollutant and the photocatalyst. Another likely contributor to the increased activity is from the stable immobilisation of the TiO_2_ nanoparticles on the polymer matrix (Figure 2), in contrast to the 13% loss of the particles observed in the previous study [28]. 

Similarly to the MB case, 95% of degradation was achieved for CIP after 240 min (Figure 4b); a significantly lower 50% degradation was observed for IBP after 300 min (Figure 4c). 

The adsorption and degradation fractions, as well as apparent reaction rates for the 1st and 4th use cycles of the TiO_2_/PVDF-TrFE photocatalyst, are summarised in Table 1. The higher adsorption fraction after the fourth use, observed in all the pollutants, is related to the increasing pore interconnectivity (pores inside the pores, as shown in Figure 1c), which is in agreement with the pore size reduction observed in the membrane after use (from 25–325 to 5–65 µm). In this context, the high adsorption of IBP onto the nanocomposite likely explains the increased (from 48% to 66%) photocatalytic efficiency during the last cycle (Figure 4c). The degradation reaction rate slightly decreases for MB from the first to the forth use cycle, while for CIP the reaction rate increases from the first to the fourth use. For IBP, the rate variations with the number of use cycles are negligible despite the significant degradation efficiency; this is caused by tendency inversion (all the kinetic fits are available in Supplementary Information—Figure S2–S4). The fact that MB has a higher affinity to the PVDF-TrFE, as proved in control (Figure 4a), explains the reduction of the reaction rate in the fourth use because of the saturation of the polymer reached in the previous uses. In this way, the following tests will present slower kinetics for MB removal and consequently, a reduced degradation rate. 

Table 2 shows the details of other works with immobilised nanocatalysts. Because of the different experimental conditions and measurements of the photocatalytic performance, comparisons are not straightforward. This kind of report allows contextualising of our results.

Concerning MB, the presented works report good efficiencies under UV radiation (90% in 360 min) using a highly porous substrate. However, UV radiation has almost twice the intensity of the one we used, and the efficiency is below the one we obtained (100% in 240 min). 

CIP degradation literature is exemplified with TiO_2_/montmorillonite (MMT) and TiO_2_/kaolinite nanocomposites because any polymeric nanocomposite was found to address CIP degradation. These materials do present interesting degradation efficiencies. Even though the TiO_2_ is immobilised in a substrate, this nanocomposite is used in suspension, which implicates the use of expensive and time-consuming filtration or separation processes to reuse them. The results obtained for TiO_2_/kaolinite, using a UV intensity six times higher the one we used, give even more significance to our material efficiency. 

IBP degradation, under visible radiation, for polyacrylonitrile (PAN)/carbone nanotubes (CNT)/TiO_2_-NH_2_ presents high degradation rates, but the authors didn’t account with the high adsorption properties of CNT on the controls, and the poly(acrylic acid) (PAA) /poly(allylamine hydrochloride) (PHA)/TiO_2_ nanocomposite lacks reusability tests. 

Overall, compared with Table 2, our results are not very different, especially if we stress that some of the presented works use higher radiation intensities or nanocomposites for suspended photocatalysis. Our nanocomposite exhibits an exciting trade-off between reusability and efficiency, which is even more relevant when the range of applications is enlarged to several contaminants. 

## 4. Conclusions

In this work, a TiO_2_/PVDF-TrFE microporous nanocomposite system previously reported as a promising photocatalyst was optimised and tested against two novel compounds representative of real common pharmaceutical pollutants: an antibiotic (CIP) and an anti-inflammatory drug (IBP). The optimisation of the nanocomposite targeted two main areas: reducing by roughly one-half the concentration of the TiO_2_ photocatalyst nanoparticles to increase cost-effectiveness and improving the characteristics of the TiO_2_/PVDF-TrFE nanocomposite at the microstructure level to enhance the photocatalytic efficiency. Specific microstructure improvements included the increased pore sizes and more robust immobilisation of the TiO_2_ nanoparticles onto the polymer matrix. In a direct comparison using photocatalytic degradation of MB, the enhanced efficiency (achieving a complete degradation of MB) of the optimised TiO_2_/PVDF-TrFE nanocomposite has been confirmed, indicating that the selected optimisation parameters have been both appropriate and effective. Furthermore, 95% of degradation was achieved for CIP, proving that the efficiency, stability, and reusability demonstrated with MB can be extended to a model pharmaceutical pollutant. The results also indicate that no nanoparticle leaching occurs, as no significant photocatalytic efficiency changes are detected after four cycles—proper attachment of nanoparticles to the microporous polymer matrix. 

From tests with IBP, despite the achieving ~60% pollutant degradation, possible physicochemical parameters have been identified for future optimisation of the TiO_2_/PVDF-TrFE nanocomposite system to address a broader range of emerging pollutants.

## Figures and Tables

**Table 1:** Comparison of the photocatalytic degradation and reaction rate (k) of MB, CIP, and IBP by the TiO_2_/PVDF-TrFE microporous nanocomposite, according to the first and last use.

	Adsorption (%)	Degradation (%)	k (min ^−1^)	R^2^
MB				
1st use	13	99	0.019	0.9669
4th use	15	97	0.015	0.9957
CIP				
1st use	7	93	0.01	0.9934
4th use	17	98	0.02	0.9818
IBP				
1st use	3	48	0.003	0.9641
4th use	22	66	0.003	0.9437

**Table 2:** Comparison of results with related works that used immobilised nanocatalysts on the degradation of MB, CIP, and IBP.

Pollutant	Material	TiO_2_	Radiation	Quantity (mg L^−1^)	Degradation (%)	Time (min)	Ref
MB	TiO_2_/HPDE	-	UV (100 W)	1.0 × 10^−5^	90	360	[59]
MB	PVDF-ZnO/Ag	4 wt.%	Visible (18 W)	10	51	100	[60]
CIP	TiO_2_/MMT	0.1 g L^−1^	UV 16 W	20	≈60	120	[61]
CIP	TiO_2_/kaolinite	0.1 g L^−1^	UV (300 W)	10	≈95	≈100	[62]
IBP	PAN-CNT/TiO_2_-NH_2_	-	Visible (125 W Xenon)	5	≈100	210	[63]
IBP	PAA/PAH/TiO_2_	In film	Sun simulador (40 W)	20	50	150	[64]

## Supplementary Materials

The following are available online at https://www.mdpi.com/1996-1944/12/10/1649/s1, Figure S1: Controls for methylene blue (MB) (a), ciprofloxacin (CIP) (b), and ibuprofen (IBP) solutions (c), Figure S2: Methylene blue (MB) degradation and kinetic fit obtained during four consecutives uses, Figure S3: Ciprofloxacin degradation and kinetic fit obtained during four consecutives uses, Figure S4: Ibuprofen degradation and kinetic fit obtained during four consecutives uses.
