# Supplementary material for: Photocatalytic Microporous Membrane against the Increasing Problem of Water Emerging Pollutants
Source: Materials (Basel). 2019 May 21;12(10):1649. doi: 10.3390/ma12101649 (PMC6566406; doi:10.3390/ma12101649)
Supplement: Supplementary file 1 [file materials-12-01649-s001.pdf]

materials

Supplementary Information

Photocatalytic Microporous Membrane Against the Increasing Problem of Water Emerging Pollutants

Pedro M. Martins [1,2,3,*,†], Joana M. Ribeiro [1,†], Sara Teixeira[4], Dmitri. Y. Petrovykh [5], Gianaurelio Cuniberti [4,6,7], Luciana Pereira [2], and Senentxu Lanceros-Méndez [1,8,9,*]

1 Centre of Physics, University of Minho, 4710-057 Braga, Portugal; joanaribeiro93@hotmail.com
2 Department of Biological Engineering, University of Minho, 4710-057 Braga, Portugal; lucianapereira@deb.uminho.pt
3 Institute of Science and Innovation for Bio-Sustainability (IB-S), University of Minho, 4710-057 Braga, Portugal
4 Institute for Materials Science and Max Bergmann Center of Biomaterials, TU Dresden, 01062 Dresden, Germany; sara.teixeira@nano.tu-dresden.de (S.T.); g.cuniberti@tu-dresden.de (G.C.)
5 International Iberian Nanotechnology Laboratory, Avenida Mestre José Veiga, 4715-330 Braga, Portugal; dmitri.petrovykh@inl.int
6 Dresden Center for Computational Materials Science, TU Dresden, 01062 Dresden, Germany;
7 Center for Advancing Electronics Dresden, TU Dresden, 01062 Dresden, Germany
8 Basque Center for Materials, Applications, and Nanostructures, UPV/EHU Science Park, 48940 Leioa, Spain
9 IKERBASQUE, Basque Foundation for Science, 48013 Bilbao, Spain
* Correspondence: pamartins@fisica.uminho.pt (P.M.M.); lanceros@fisica.uminho.pt (S.L.-M.)
† Pedro M. Martins and Joana M. Ribeiro contributed equally to this work

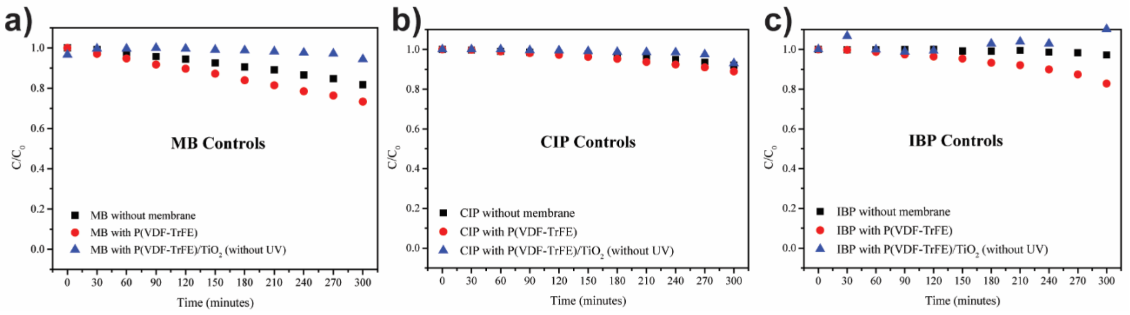

Figure S1. For methylene blue (MB) (a), ciprofloxacin (CIP) (b), and ibuprofen (IBP) solutions (c).

Figure S2, S3, and S4 shows the kinetic fits for methylene blue (MB), ciprofloxacin (CIP), and ibuprofen (IBP), respectively.

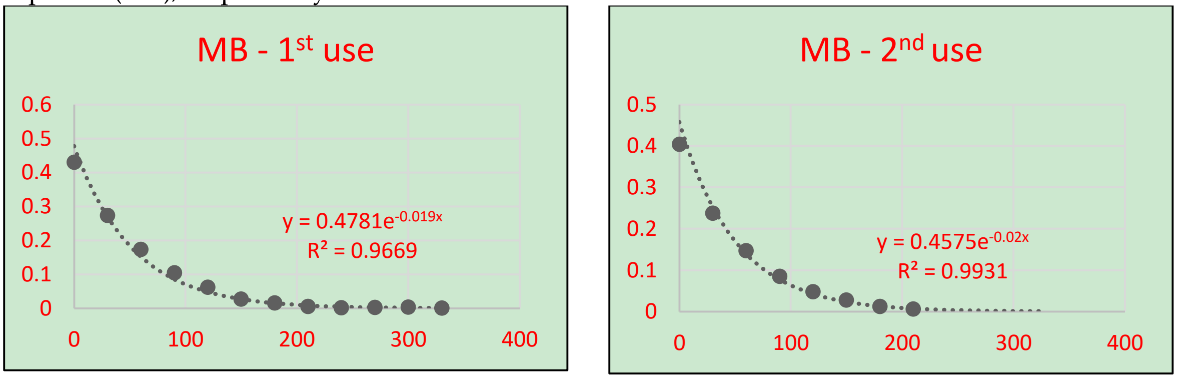

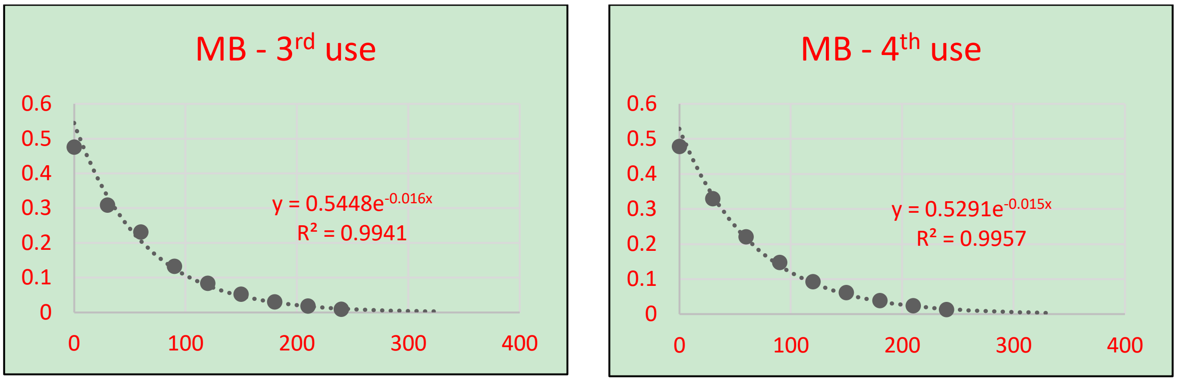

Figure S2. Blue (MB) degradation and kinetic fit obtained during four consecutives uses.

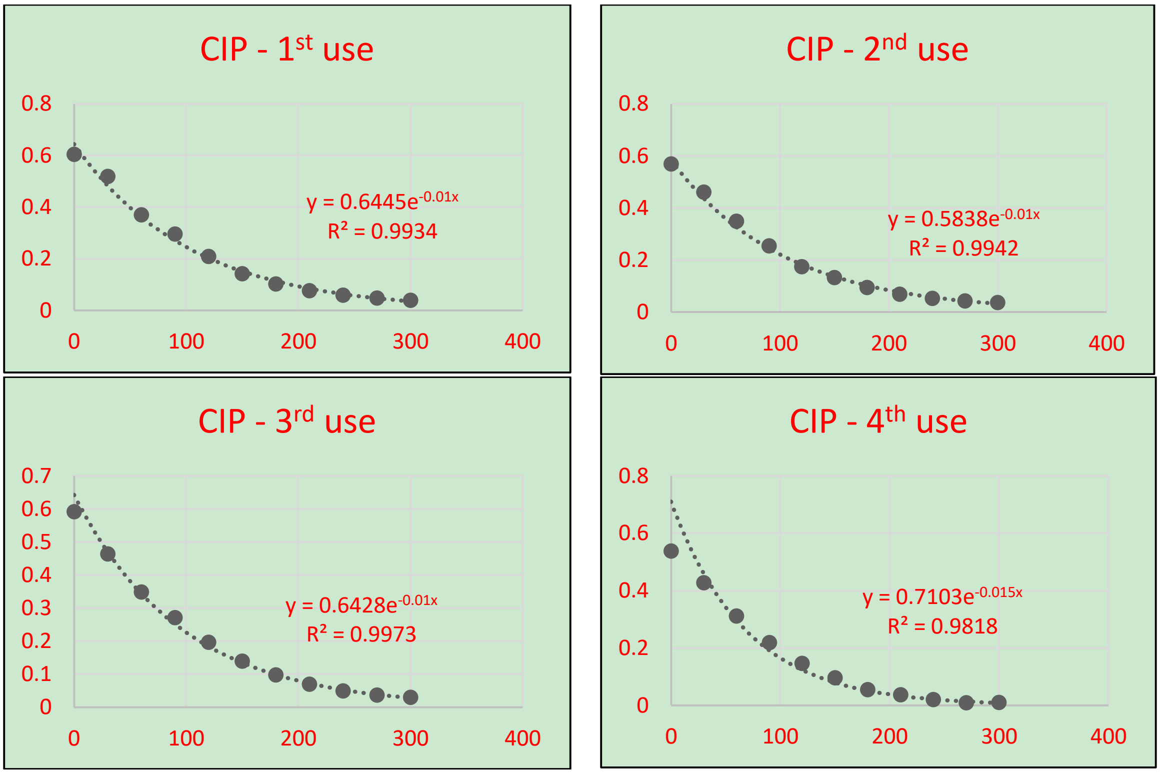

Figure S3. Degradation and kinetic fit obtained during four consecutives uses.

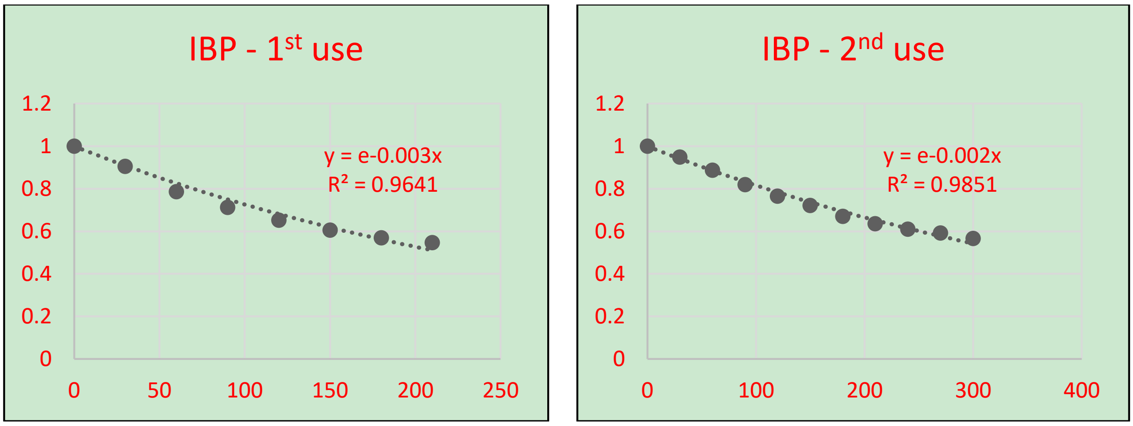

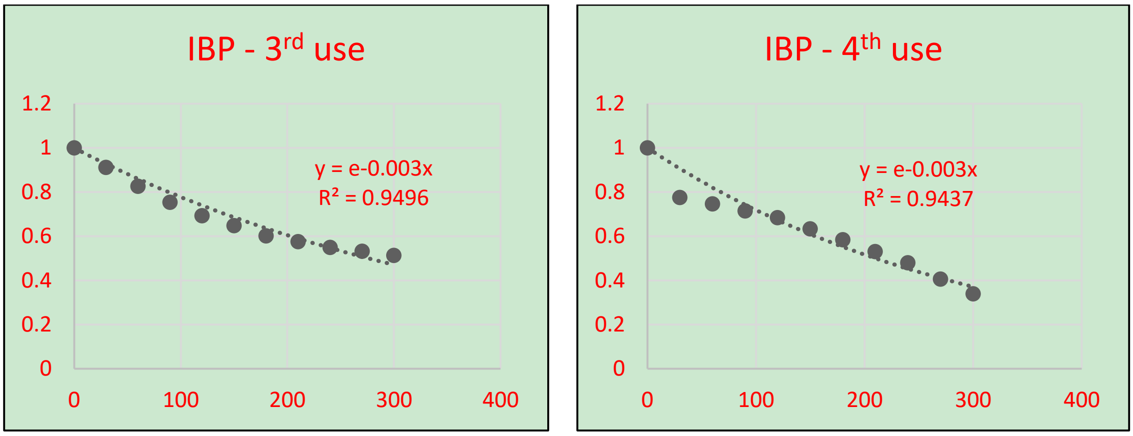

Figure S4. Degradation and kinetic fit obtained during four consecutives uses.
